# Bidirectional scaling of vocal variability by an avian cortico‐basal ganglia circuit

**DOI:** 10.14814/phy2.13638

**Published:** 2018-04-24

**Authors:** Jonathan B. Heston, Joseph Simon, Nancy F. Day, Melissa J. Coleman, Stephanie A. White

**Affiliations:** ^1^ Interdepartmental Program in Neuroscience University of California Los Angeles California; ^2^ Undergraduate Interdepartmental Program for Neuroscience University of California Los Angeles California; ^3^ Department of Integrative Biology and Physiology University of California Los Angeles California; ^4^ W. M. Keck Science Department of Claremont McKenna College Pitzer College, and Scripps College Claremont California

**Keywords:** Birdsong, chemogenetics, motor control, variability, vocalization, zebra finch

## Abstract

Behavioral variability is thought to be critical for trial and error learning, but where such motor exploration is generated in the central nervous system is unclear. The zebra finch songbird species offers a highly appropriate model in which to address this question. The male song is amenable to detailed measurements of variability, while the brain contains an identified cortico‐basal ganglia loop that underlies this behavior. We used pharmacogenetic interventions to separately interrogate cortical and basal ganglia nodes of zebra finch song control circuitry. We show that bidirectional manipulations of each node produce near mirror image changes in vocal control: Cortical activity promotes song variability, whereas basal ganglia activity promotes song stability. Furthermore, female conspecifics can detect these pharmacogenetically elicited changes in song quality. Our results indicate that cortex and striatopallidum can jointly and reciprocally affect behaviorally relevant levels of vocal variability, and point to endogenous mechanisms for its control.

## Introduction

Bowling, darts, or the basketball free throw could seemingly be mastered by simply determining the movement that produces a strike, bulls‐eye, or basket and repeating it in a stereotyped manner; yet motor variability ensures that perfect scores in each of these sports is rare. Rather than being the simple product of noise, variable motor performance is hypothesized to be actively generated by the nervous system in order to facilitate motor learning (Sutton and Barto 1998). Indeed, such variability positively predicts procedural learning in a variety of tasks and species including humans (Kerr and Booth 1978). The neural mechanisms underlying the generation of variability, however, are incompletely characterized.

**Figure 1:**
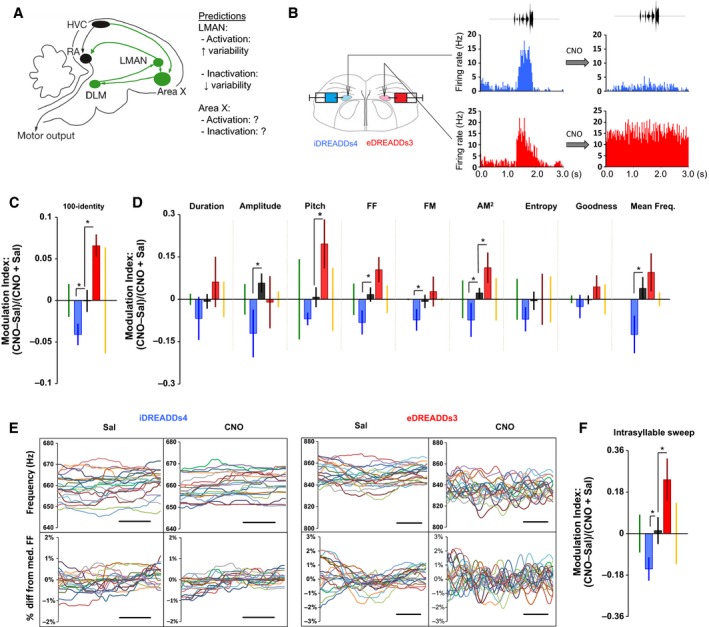
Validation of methodology in LMAN confirms it is a positive regulator of vocal variability. (A) The zebra finch brain contains two interconnected pathways controlling vocal motor output. One is the posterior vocal‐motor pathway (black) consisting of the cortical nuclei HVC (used as a proper name) and the robust nucleus of the arcopallium (RA), and brain stem motor neurons that control vocal output. This pathway is essential for the execution of learned vocalizations. The second pathway is the anterior forebrain pathway (green), a song‐dedicated cortico‐basal ganglia loop, consisting of HVC, the striato‐pallidal nucleus Area X, the dorsolateral thalamic nucleus (DLM), and the cortical nucleus LMAN which sends bifurcating axons to both Area X as well as RA, thereby allowing this pathway to affect vocal output. Area X is predominantly composed of striatal medium spiny neurons and also contains pallidal interneurons and projection neurons. It is well established that LMAN positively regulates vocal variability but it has been unclear whether Area X plays a role in controlling variability. (B) Three birds were unilaterally injected in LMAN with each DREADDs type. A CNO‐elicited decrease in multiunit activity was observed in two of three iDREADDs4 injected hemispheres, whereas increased activity was observed in two of three eDREADDs3 injected hemispheres. (No change was observed in the other two hemispheres.) Shown here is neural activity in response to a bird's own song play back recorded in LMAN in a bird injected with iDREADDs4 in one hemisphere (blue) and eDREADDs3 in the other (red). Following administration of CNO, neural activity is attenuated on the iDREADDS4 injected side and enhanced on the eDREADDs3 side. (C) Intersyllable variability of song syllables as measured by syllable identity either decreases following inhibition (via iDREADDs4; blue; *n *=* *6) or increases following excitation (via eDREADDs3; red; *n *=* *4) of LMAN. A positive MI score (ordinate axis) reflects increases in variability for this and all other measures of variability. (See Materials and Methods for explanation of the MI). For each measure shown here, in (E and F) below, and similar panels in Fig. 3, ethologically relevant windows of variability are indicated by green (social context‐dependent) or orange (practice‐induced) bars which flank values for iDREADDs4 (blue), control (black) and eDREADDs3 (red), respectively. Asterisks indicate *P *<* *0.05 relative to controls using one‐tailed unpaired resampling tests throughout. (D) CNO‐induced activation of iDREADDs4 versus eDREADDs3 in LMAN produces bidirectional changes in intersyllable variability across multiple feature‐specific measures of variability as represented by the coefficients of variation (CV). (E) Traces show raw (top) and demeaned FF (bottom) contours from 20 renditions of a representative flat harmonic syllable. Inhibition of LMAN via CNO‐induced activation of iDREADDs4 leads to a convergence and flattening of demeaned FF traces relative to the saline condition; intrasyllable fluctuations in FF are thereby reduced. Conversely, excitation of LMAN via CNO‐induced activation of eDREADDs3 leads to a divergence and increase in the fluctuations. Scale bar = 10 msec. (F) Similar to intersyllable variability, the intrasyllable sweep shows positive, bidirectional control of intrasyllable variability. LMAN, lateral magnocellular nucleus of the anterior nidopallium; DREADDs, designer receptors exclusively activated by designer drugs; CNO, clozapine *N*‐oxide.

The zebra finch song control system offers an opportunity to dissect these mechanisms. The zebra finch brain contains a vocal‐dedicated cortico‐basal ganglia loop known as the anterior forebrain pathway (AFP; Fig. 1). The AFP receives excitatory input from and sends excitatory output to a premotor pathway required for song production. Within the AFP, increasing activity within the cortical lateral magnocellular nucleus of the anterior nidopallium (LMAN) increases song variability, whereas lesioning LMAN or decreasing its activity decreases song variability (Woolley and Kao 2015). These observations suggest that LMAN functions as a “variability injector,” but because it is the output of a larger circuit, it is unclear whether this variability is intrinsic to LMAN or inherited from elsewhere.

One alternative source of variability is the basal ganglia song control nucleus, Area X, which is composed of both striatal and pallidal cell types (Carillo and Doupe 2004). Area X is upstream of LMAN in the AFP circuit and also receives a recurrent LMAN projections (Vates et al. 1997). Lesions of Area X yield transient or inconsistent effects on song variability (Goldberg and Fee 2011; Kojima et al. 2013), questioning the role of this basal ganglia node in motor variability. Other evidence, however, indicates that Area X mediates changes in vocal variability. For example, the instantaneous stabilization of song that occurs when a male bird performs to a female requires dopamine 1 (D1) receptor activation in Area X (Leblois et al. 2010). Indeed, social context induced changes in Area X spiking activity suggest it is the neural nexus mediating this transition (Woolley et al. 2014). Moreover, manipulations of the language‐related gene *FoxP2* within Area X interfere with rapid social context‐dependent (Murugan et al. 2013) and slower practice‐dependent (Heston and White 2015) changes in variability.

These observations raise the possibility that Area X contributes to the control of vocal exploration that can be revealed on a relatively short time scale. Here, we test this hypothesis using viral‐driven expression of designer receptors exclusively activated by designer drugs (DREADDs) to transiently, bidirectionally, and independently alter the activity of LMAN or Area X neurons and measure the effect on song.

## Materials and Methods

### Animals

All animal use was in accordance with NIH guidelines for experiments involving vertebrate animals and approved by the University of California Los Angeles Chancellor's Institutional Animal Care and Use Committee and were consistent with the American Veterinary Medical Association guidelines. Birds were obtained from our breeding colony, and housed in climate‐controlled rooms inside cages and aviaries with a 13:11 lights on:lights off cycle including half hours of dawn and dusk. Birds had unlimited access to food, grit and water and were provided nutritional supplements and environmental enrichments.

### Behavior

Adult male birds (100 + days posthatch) were moved to sound attenuation chambers (Acoustic Systems, Austin, TX) and allowed to acclimate for several days. Thereafter, procedures differed depending on whether the bird received herpes simplex virus (HSV), which reaches peak expression after several days, or adeno‐associated virus (AAV) which reaches peak expression after ~3 weeks. Initial experiments were performed using an HSV, which drove DREADDs expression off of a nonspecific mCMV promoter. These results were confirmed using an AAV which included the CaMKII promoter to ensure expression within the major LMAN (Jones et al. 1994; Nathanson et al. 2009) and Area X (Hein et al. 2007) cell types (see below). For HSV, birds underwent song recording following presurgery administration of saline on day 1 and clozapine *N*‐oxide (CNO) on day 2. On day 3, they were stereotaxically injected with HSV as described below. Following surgery, birds recovered in sound attenuation chambers for 1–2 days, and then underwent postsurgery behavioral testing. For AAV, birds first underwent surgery to inject virus as described below. After 3–4 weeks, the bird was habituated by IP injection of saline. The following day it was administered either saline or CNO and then 2 days later was administered CNO, or saline, respectively.

Sexually mature but inexperienced female zebra finches were used to examine conspecific responses to males’ songs as described under female preference testing, below.

Adequate sample size was estimated based on power analysis and prior studies that examined changes in male song variability (LMAN: Ölveczky et al. 2005 – six birds; Hamaguchi and Mooney 2012 – three birds; Leblois et al. 2010 – four birds. Area X: Miller et al. 2010 – 7–10 birds; Heston and White 2015 – 7–10 birds; Goldberg and Fee 2011 – 7–12 birds; Burkett et al., 2018 – 7 birds). We thus used 7–9 birds per DREADDs construct (inclusive of HSV and AAV) for Area X experiments. Each bird was considered to be both a biological and technical replicate. No data were considered outliers and therefore no data were excluded on a statistical basis.

Stereotaxic neurosurgery was performed as in Heston and White (2015) with the following modifications: LMAN was targeted at a site +5.15 mm anterior and +1.6–7 mm lateral to the bifurcation of the midsagittal sinus at a depth of 2.0 mm. Birds were excluded from behavioral analysis if virus was found to be mistargeted or retrogradely transfected to afferent nuclei, or if a large lesion was identified at the injection site. Only two birds were excluded on the basis of off‐targeting of the virus.

### Viruses

#### HSV

All HSV was obtained from the MIT Viral Core stock virus catalog at a titer of 7 × 10^8^ IU/mL. Behavioral experiments were conducted on birds that were injected with the following viruses each of which drive expression off of the mCMV promoter: ST HSV‐hM4Di‐mCherry, ST HSV‐hM3Dq‐mCherry, and ST HSV‐mCherry. All HSV was diluted to 60–75% with saline before injection into the brain to avoid neurotoxicity and retrograde trafficking. Two birds were injected in LMAN with HSV‐iDREADDs4, three birds were injected in LMAN with HSV‐iDREADDs3, three birds were injected in Area X with HSV‐iDREADDs4, and two birds were injected in Area X with HSV‐eDREADDs3.

#### AAV

Custom designed AAVs (serotype 1) were produced by Virovek (Hayward, CA; Heston and White 2015) containing either iDREADDs4 or eDREADDs3 driven off of the α‐CaMKII promoter (AddGene). Both viruses had a titer of 1 × 10^13^ vg/mL and were injected at a volume of 500 nL. Two birds were injected in LMAN with CaMKII‐AAV ‐iDREADDs4, three birds were injected in LMAN with CaMKII‐AAV‐iDREADDs3, six birds were injected in Area X with CaMKII‐AAV‐iDREADDs4, and five birds were injected in Area X with CaMKII‐AAV‐eDREADDs3, all from Virovek. The α‐CaMKII promoter was chosen because, based on other species, it was predicted to express exclusively in LMAN excitatory neurons (Jones et al. 1994; Nathanson et al.^2009^) and Area X MSNs given that these are the only neurons in this nucleus which express α‐CaMKII (Hein et al. 2007). In addition, vectors encoding DREADDs were obtained from the UNC Vector Core (University of North Carolina, Chapel Hill, NC). The following vectors were used: AAV5‐CaMKIIα‐HA‐hM3D(Gq)‐IRES‐mCitrine (two Area X injected birds) and AAV5‐CaMKIIα‐HA‐hM4D(Gi)‐IRES‐mCitrine (two Area X injected birds).

### CNO and saline administration

Both before and after surgery, birds were administered once with CNO (150 μL at 0.1 mg/mL, i.p.; given ~15 g average weight of an adult zebra finch this is equivalent to 1 mg/kg body weight; Sigma–Aldrich, St. Louis, MO) and once, on a different day, with saline (150 μL). After each procedure, the bird was distracted from singing for 1 h to prevent singing‐induced changes in song variability (Miller et al. 2010) and allow for CNO activation of DREADDs. Songs were recorded and analyzed as previously described (Heston and White 2015).

### Intersyllable variability analysis

The CNO‐dependent change in variability was expressed as the modulation index (MI) which followed the formula (CNO CV − Sal CV)/(CNO CV + Sal CV). Thus, if administration of CNO led to an increase in variability relative to administration of saline, the MI would be positive, but would be negative if CNO decreased variability. If CNO was without effect, as we expected in control birds, the MI will be at or near zero. Other measures of variability were evaluated by replacing the CV in the formula with metrics such as identity or sweep (see below).

### Intrasyllable variability analysis

Analysis of intrasyllable fluctuations in FF was limited to flat or near flat syllables or subsyllable elements. A custom‐written software program (NFD) was used to track FF across a syllable. A region of interest (ROI) was defined within the flat portion of a syllable (minimum of 30 msec long) and the FF was tracked at each millisecond. Several birds were excluded from this analysis because their songs did not contain a syllable with an ROI that met our criteria.

Three measures were used to quantify variability across the ROI. The first was to measure the intrasyllable coefficient of variation (CV) and was obtained by calculating the CV of all the 1 msec bins across the ROI. The second measure, which is operationally referred to as sweep, is proportional to the cumulative change in FF across the syllable. First, each FF string was demeaned and divided by the intrasyllable median FF so that each 1 msec time bin was expressed as a percent deviation about the median. Sweep was defined as **Σ**(X − [X − 1])^2^ where X represents the percent deviation at a given 1 msec time bin and X − 1 is the percent deviation at the preceding bin. The final measure of intrasyllable variability was template variability which compared an individual syllable's fluctuations around a typical or template version of that syllable. This was calculated by obtaining 20 renditions and calculating the median percent deviation about the intrasyllable median at each time bin. The resultant median trajectory of the FF trace formed a template against which each individual trace could be measured. Template variability was defined as **Σ**(X − X_t_)^2^ where X is the percent deviation from the median FF of the template at each millisecond and X_t_ is the percent deviation of the template at that millisecond. These three measures were obtained for 20 renditions of a syllable and were represented by the median.

### Ethological range of variability

To obtain ethologically relevant frames of reference for the magnitude of changes in variability induced here by DREADDs, we reanalyzed and replotted data from previous publications concerning natural levels of variability. Specifically, we determined the average change in song variability as a function of social context (directed singing [D] vs. undirected singing [UD]) or vocal practice (following nonsinging [NS] vs. UD singing). The social context‐induced change was obtained from unmanipulated birds in Miller et al. (2015). In this case, social context induced change in variability was calculated as (D − UD)/(D + UD). As with the main results, the syllable changes were averaged within birds and each bird was treated as a single data point. These were then averaged for each measure of variability yielding a typical social context‐induced change. The practice‐induced change in variability was obtained using data from control birds in Heston and White (2015) and followed identical procedures but used the formula (UD − NS)/(UD + NS) where UD represents the variability after 2 h of UD and NS represents the variability after 2 h of NS. In both cases, the averages were plotted bidirectionally as in neither case could one state be defined as the baseline.

### Histological methods

To examine the efficacy in targeting and expression of viral injections, birds were perfused with warm saline followed by ice cold 4% paraformaldehyde in 0.1 mol/L phosphate buffer, and their brains extracted for histological analysis. Characterization of viral transfection was carried out using immunohistological methods described by Miller et al. (2008). AAV‐driven expression of mCherry required immunostaining with an anti‐dsRED antibody (#632496; Takara Bio USA Inc, Mountain View, CA) for visualization. HSV‐driven expression could typically be visualized without an immunostaining experiment (indicative of greater transduction levels than those achieved with AAV) but one was often done to boost native fluorescence. Brains were sectioned on a crysostat (Leica Microsystems, Bannockburn, IL) at a thickness of 30 μm.

### *In vivo* electrophysiology recording

*In vivo* multiunit recordings were performed as described in Williams et al. (2012). Briefly, birds were anesthetized and a small craniotomy was made over the approximate location of LMAN and a carbon fiber electrode (Kation Scientific, Minneapolis, MN) was lowered into the brain with a micromanipulator. All recordings were amplified (A‐M systems, Sequim, WA), filtered (300 Hz high pass, 5 kHz low pass), digitized at 20 kHz (Micro1401, CED, Cambridge, England), and collected using Spike 2 software (CED). For each recording, 20 to 40 repetitions of the bird's own song were played with a 10 sec interstimulus interval. LMAN, as well as several other song system nuclei, is sensitive to playback of the bird's own song (BOS). Therefore, BOS playback was used to elicit a baseline auditory response from which any DREADDS modulation of neural activity could be detected. After obtaining a stable baseline, the bird was intraperitoneally administered CNO as described for behavioral experiments. After each recording session, electrolytic lesions (+10 μA for 10 sec) were made at the LMAN recordings site to enable histological confirmation of the recording location.

### Female preference testing

Male songs were evaluated by a cohort of eight, sexually mature but inexperienced female birds that had been group housed in sound attenuation chambers. For each session, calls were counted from individually housed females during a 10 min baseline period that was followed by five 15 min “playback‐response” periods. Each female heard only one male's song per session, which included three playback periods of one song context and two of the other context (e.g., three Sal, two CNO or three D, two UD, etc.,) in a pseudorandom order. Each “playback‐response” period consisted of 5 min of song playback in which bouts of song recorded in a single behavioral context were played at 10 sec intervals. This 5 min playback period was followed by a 10 min response period in which no songs were played back. For example, a female could listen to the song of an individual male bird that was injected in Area X with eDREADDs3 in the order: Baseline (silence)–CNO–Sal–Sal–CNO–Sal. Changes in calling behavior evoked by each song type were quantified using the formula ln((post + 1)/(pre + 1)). From this, the median change in calling was calculated to yield a calling index for each song type. The CNO‐ or directed song‐induced change was calculated as (CNO index–Sal index) or (D index–UD index). Sessions were excluded if the female failed to call in four or more of the six epochs in a session or failed to call at least 30 times across the six epochs (one baseline and five response periods). Each male's song was evaluated by four of the eight females and the CNO‐induced change in female response was derived from the median of those four sessions.

### Statistics

Resampling statistics were used throughout our study because we had no a priori expectation of normality in the data set. (Resampling statistics makes no assumptions about the distribution of data but rather creates an unbiased null distribution derived from the observed data.) The unpaired test begins by calculating the difference in‐group means. This value represents the test statistic, M. We then created pseudo‐data sets with same N as the actual group sizes and randomly drawn with replacement from a combined set of actual data points. This process was repeated 10,000 times, keeping track of pseudo‐M values. These values formed the distribution of M under the null hypothesis, reflecting the values of M we could have expected if the direction if the distribution of data points was random, and was not an effect of the experimental paradigm. Finally, the number of pseudo‐Ms that was as large, or larger, than the actual M was determined and this number divided by 10,000. This value reflects the reported *P*‐value.

Our first statistical test compared the modulation index of iDREADDs4 versus eDREADDs3 for each measure using a two‐tailed a comparison of two groups using resampling. If a significant effect was found, a one‐tailed test was used to compare each group to control. Because the data in Figure 3 tended to generate large outliers, the data were displayed and statistically tested using the median. Briefly, the data were represented using the median, and error bars represent the median absolute data about the median, divided by the square root of the N. A resample test was used to test if the distribution of M overlapped with 0.

For the female preference section, we also include Cohen's d’ which is a measure of effect size and is calculated as the difference between the means of two samples divided by the pooled standard deviation of the two samples.

### Validation of pharmacogenetic approach

Yazaki‐Sugiyama et al. (2015) pioneered the use of ivermectin‐sensitive chloride channels in zebra finches as a pharmacogenetic approach to silence RA neurons. To our knowledge, however, the use of DREADDs has not been previously reported in the song control system. To test their efficacy, adult birds were injected bilaterally in either LMAN or Area X with a virus that expressed either eDREADDs3 to increase neural activity or iDREADDs4 to decrease neural activity (Armbruster et al. 2007) following administration of the DREADDs ligand clozapine‐N‐oxide (CNO). For the majority of experimental birds (*n *=* *16), an AAV that utilized the α‐CaMKII promoter (CaMKII‐AAV) was used to drive receptor expression in LMAN excitatory projection neurons (see Materials and Methods; Jones et al. 1994; Dittgen et al. 2004) or Area X medium spiny neurons (MSNs; Hein et al. 2007). A minority of experimental birds (*n *=* *10) were injected with a HSV that utilized a nonspecific promoter. In each case, the DREADDs construct was covalently linked to the fluorophore mCherry or mCitrine to enable visualization.

We verified that these viruses targeted relevant cell types in each nucleus (excitatory projection neurons in LMAN or medium spiny neurons in Area X; Fig. S1) and that they could be used to alter neural activity in the predicted manner (Fig. 1B). To assess how these viral manipulations altered song behavior, syllable variability was measured following systemic administration of CNO on a given day and compared to that following saline (SAL) administration on a different day in order to obtain the modulation index (MI). Control song syllables were obtained from birds that had not undergone surgery (*n *=* *14) and from birds injected with virus encoding mCherry alone (*n *=* *3). Three types of variability were assessed: rendition‐to‐rendition (intersyllable) differences, moment‐to‐moment (intrasyllable) fluctuations in fundamental frequency (FF), and alterations in syllable sequencing (syntax).

## Results

### LMAN acts as a variability injector

As a first behavioral validation of our methodology, we examined intersyllable song variability, which LMAN is well established to positively regulate (Woolley and Kao 2015). As predicted, activation of eDREADDs3 (*n *=* *4) in LMAN projection neurons increased syllable variability, whereas activation of iDREADDs4 (*n *=* *6) led to a decrease across numerous measures of intersyllable variability (Fig. 1C and D). This effect was similar between HSV and CaMKII‐AAV injected birds (Fig. S2) and altered the variability of syllable features, as measured by their coefficients of variation (CV), but not the means of those features.

Intrasyllable variation as fluctuations of FF was also measured. This type of variability is thought to represent ultrafast motor exploration and can be used to guide learning on a millisecond timescale (Charlesworth et al. 2011), but its neural underpinnings remain unexplored. Similar to intersyllable variability, LMAN positively and bidirectionally regulated this type of variability. Activation of eDREADDs3 increased FF fluctuations, whereas activation of iDREADDs4 decreased each of the three different measures of intrasyllable variability (Fig. 1E and F). Interestingly, this effect was driven primarily by HSV injected birds (Fig. S3). There was no effect of LMAN manipulations on syntax variability (data not shown).

### Area X can bidirectionally affect song variability

**Figure 2:**
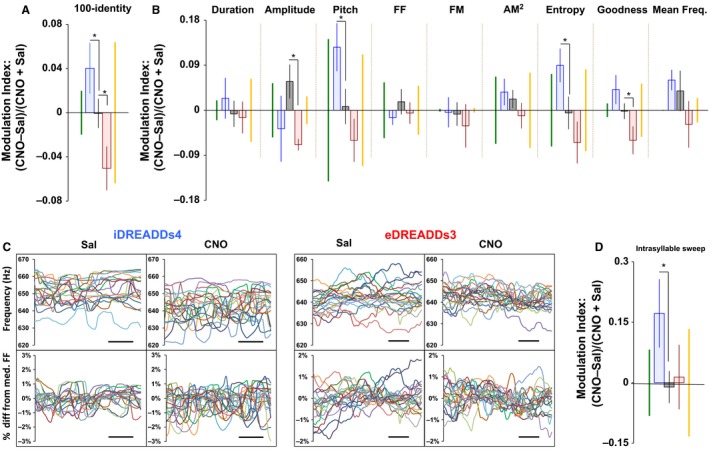
Area X negatively regulates intersyllable and unidirectionally affects intrasyllable variability. (A) Intersyllable variability of song syllables as measured by syllable identity increases or decreases following inhibition or excitation of Area X, respectively. For iDREADDs4 (*n *=* *9), 100‐identity: *P *=* *0.046; identity: *P *=* *0.013. For eDREADDs3 (*n *=* *7), 100‐identity: *P *=* *0.016; identity: *P *=* *0.023. (B) CNO‐induced activation of iDREADDs4 versus eDREADDs3 in Area X produces bidirectional changes in intersyllable variability across multiple feature‐specific measures. For iDREADDs4, CV of pitch: *P *=* *0.029; CV of entropy: *P *=* *0.034. For eDREADDs3, CV of amplitude: *P *=* *0.014; CV of pitch goodness: *P *=* *0.030. (C) Traces show raw (top) and demeaned FF (bottom) contours from 20 renditions of a representative flat harmonic syllable. Inhibition of Area X via CNO‐induced activation of iDREADDs4 led to an increase in intrasyllable FF fluctuations. Excitation of Area X via CNO‐induced activation of eDREADDs3 had no apparent effect on these fluctuations. Scale bar = 10 msec. (D) Similar to intersyllable variability, CNO‐induced activation of iDREADDs4 in Area X led to enhanced intrasyllable variability (Intrasyllable sweep: *P *=* *0.039; intrasyllable CV of FF: *P *=* *0.014). Unlike previous measures, however, CNO‐induced activation of eDREADDs3 was without effect. DREADDs, designer receptors exclusively activated by designer drugs; CNO, clozapine N‐oxide.

These analyses were then applied to syllables produced following manipulations of Area X, because its role in variability generation is less clear. To our surprise, Area X regulated variability in a manner reciprocal to LMAN. Activation of iDREADDs4 (*n *=* *9) on Area X MSNs increased numerous measures of intersyllable variability, whereas activation of eDREADDs3 (*n *=* *8) decreased intersyllable variability (100‐identity: *P *=* *0.046; identity: *P *=* *0.013; Fig. 2A and B), albeit these changes were limited in magnitude compared to LMAN manipulations. This effect was similar between viral types (Fig. S4). Moreover, unlike LMAN, Area X manipulations extended beyond the CVs of song features to affect the mean of one feature: syllable entropy. Activation of eDREADDs3 decreased average syllable entropy (i.e., resulted in greater syllable structure), whereas activation of iDREADDs4 led to a nonsignificant increase in entropy (data not shown).

Interestingly, activation of iDREADDs4 in Area X enhanced intrasyllable variability (intrasyllable CV of FF: *P *=* *0.018), but activation of eDREADDs3 had no effect (Fig. 2C and D). Thus, in terms of intrasyllable variability, Area X acted as a unidirectional variability suppressor. As with LMAN, this effect on intrasyllable structure was driven primarily by HSV injected birds (Fig. S5), and no effect on syntax variability was detected (data not shown).

### Female zebra finches perceive DREADDs‐induced changes in male vocal variability

**Figure 3:**
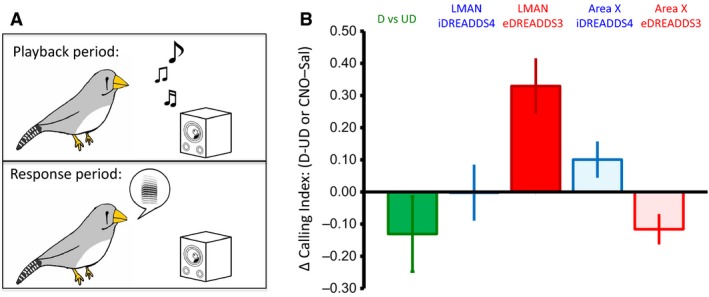
DREADDs‐induced changes in song are detected by female zebra finches. (A) Female birds heard playbacks of directed (D) versus undirected (UD) songs or of songs from DREADDS‐injected males administered saline or CNO. Songs from males were presented in five blocks of 30 playbacks. Each playback was followed by a 10‐min period of nonplayback during which females’ calls were recorded. (B) Females decreased their calling after playback of songs from males singing D song (*P *=* *0.03 two‐tailed resampling, Cohen's d’ = 0.85). Accordingly, they showed a modest decrease following playback from males in which LMAN was inhibited (*P *=* *0.13 one‐tailed resampling, Cohen's d’ = 0.12), and a robust increase when LMAN was excited (*P *=* *0.03 one‐tailed resampling, Cohen's d’ = 1.23). In line with these results, females called more following playback of songs from males in which Area X was inhibited (*P *=* *0.14, Cohen's d’ = 0.24). No change was observed following excitation of Area X (*P *=* *0.34 one‐tailed resampling, Cohen's d’ = 0.22). LMAN, lateral magnocellular nucleus of the anterior nidopallium; DREADDs, designer receptors exclusively activated by designer drugs

To determine whether these changes were perceived by conspecifics, and thus of potential ethological relevance, we tested whether female zebra finches altered their behavior in response to more stereotyped (CNO‐induced activation of Area X eDREADDs3 or of LMAN iDREADDs4) or variable (CNO‐induced activation of Area X iDREADDs4 or of LMAN eDREADDs3) songs. Females can detect minute differences in variability between an adult male's stereotyped song (referred to as D; sung by a male when courting a female) versus his more variable song (UD; sung by a male in isolation; Woolley and Doupe 2008). We assessed whether sexually naïve female birds could detect the changes in variability by counting the number of calls a female made following playback of songs elicited under different social and DREADDs‐related physiological conditions. As expected, naïve females responded differently to undirected (UD) versus directed (D) songs, demonstrating their ability to differentiate song types. In six out of the seven cases, playback of the low variability D song resulted in less calling relative to playback of more variable UD song (Fig. 3). Interestingly, this pattern of behavior was opposite to that observed in Bengalese finches by Dunning et al. (2014). Whether the difference between that study and ours reflects a species difference or subtle changes in the experimental conditions is unclear. Nevertheless, both results show that females in each species can detect differences in male song quality and change their behavior as a result.

Based on this pattern of calling, we predicted that females would call more following song playbacks from variability‐inducing manipulations that recapitulate the lower stereotypy of UD song (excitation of LMAN, inhibition of Area X MSNs) and call less following variability‐reducing manipulations (inhibition of LMAN, excitation of Area X). Indeed, females called more to song playbacks produced during excitation of LMAN, and decreased their calling to song produced during LMAN inhibition. Also in line with our prediction, song playback from males with Area X inhibition increased calling, but intriguingly Area X excitation elicited no change in calling. This pattern of results was very similar to the intrasyllable results reported above (Figs. 1F and 2D), although there were no correlations between changes in the amount of female calling and changes in male intrasyllable variability (data not shown).

## Discussion

Our major findings indicate that LMAN and Area X can act as dual regulators of vocal variability. These results reinforce the notion that LMAN positively scales vocal variability, and they provide novel evidence implicating Area X as a reciprocal regulator of variability. Moreover, both nuclei can act at the millisecond timescale suggesting a role in the moment‐to‐moment execution of motor commands. These effects were largely replicated through the use of two distinct viral constructs which produced similar changes on intersyllable variability. In contrast, changes in intrasyllable variability were only revealed with the HSV construct. This may be due to lower transduction levels achieved with the CaMKII‐AAV relative to HSV, as noted in the Materials and Methods, given that both viruses tranduced LMAN projection neurons and Area X MSNs (Fig. S1).

An additional finding is that Area X can regulate variability in a manner opposite to LMAN. This result was somewhat unexpected given that extracellular recordings and immediate early gene expression indicate that Area X, like LMAN, fires more during variable UD singing relative to D song (Woolley et al. 2014). However, this finding is consistent with the observation that D1 receptor activation in Area X increases MSN excitability and is required for social context induced changes in intersyllable variability (Ding and Perkel 2002; Leblois et al. 2010). In rats, silencing of the dorsolateral striatum increases the variability of execution of a well‐learned motor task (Rueda‐Orozco and Robbe 2015), suggesting that the output of the striatum promotes motor stability rather than motor exploration. One hypothesis that could reconcile these disparate observations is that LMAN co‐engages Area X as a negative feedback mechanism to adaptively temper and control variability. Thus, Area X would be more active when LMAN is highly active, and therefore song more variable, yet manipulations of Area X by itself would reveal it as a variability repressor.

The magnitude of DREADDs‐induced changes was smaller for Area X manipulations than for LMAN. There are a number of possibilities for this observation: Area X is larger than LMAN and therefore it is more difficult to transfect the entire nucleus; LMAN is the output of the circuit and manipulation of this nucleus may inherently lead to larger effects; and Area X has greater cellular diversity with many antagonistic connections which could dilute the effect. A caveat with our Area X results is that we do not have the data to fully understand how DREADDs manipulations affect the complex internal circuitry of Area X. Such an understanding would require single unit recordings from the many individual cell types (≥12; E.R. Fraley and S.A. White, unpubl. obs.) both before and after activation of each DREADDs type. Unfortunately, this beyond the scope of the current study and we can only speculate as to how Area X activity is affected on a cell‐type by cell‐type basis.

The mammalian striatum is composed of two antagonistic populations of MSNs, namely those of the direct and indirect pathways, which exhibit opposing effects on behavior (Lee et al. 2016). It was therefore surprising to us that nonspecific targeting of Area X MSNs by DREADDs had a net behavioral effect, rather than the effects being cancelled out. To recap, activation of MSNs by eDREADDs3 had a net effect of decreasing song variability, whereas inactivation by eDREADDs4 increased variability. This suggests that one population of MSNs dominated the other under our experimental conditions. If we assume that the function of the globus pallidus internal segment (GPi)‐like projection neurons is to inhibit the thalamus (Fig. 1) as suggested by Goldberg and Fee (2012), Goldberg et al. (2012), then our findings indicate a relative dominance of indirect pathway MSNs. In this scenario, nonspecific activation of MSNs by eDREADDs3 led to a net disinhibition of GPi‐like neurons, and decreased activity in DLM and LMAN, decreasing vocal variability. MSN inactivation by iDREADDs4 would exert the opposite effect throughout the circuit.

As with the mammalian striatum, Area X contains MSNs that form inhibitory synapses onto other MSNs as well as GPI‐like neurons which form the output of Area X (Gale and Perkel 2010). Furthermore, these neurons show differential sensitivity to D1‐ and D2‐agonists (Ding and Perkel 2002) but unlike the mammalian striatum, this exists on a continuum rather than complete segregation by receptor type. A net dominance of indirect pathway MSNs is consistent with the observed preponderance of dopamine 2 (D2) receptors relative to D1 receptors in the rodent dorsolateral striatum (Yin et al. 2009), the mammalian analog to Area X (Pfenning et al. 2014).

Alternatively, if we assume that the function of GPi‐like neurons is to disinhibit the thalamus as suggested by Person and Perkel (2005, 2007), then the entire scenario would reverse. Future work aimed at targeting DREADDs expression to the indirect versus the direct pathway of MSNs may be critical in dissecting each role in vocal variability. Currently, this may not be as feasible in songbirds as in rodents (Lee et al. 2016), given that both D1 and D2 receptors can be expressed on a single Area X MSN (Ding and Perkel 2002). In any case, the use of naturalistic activity to engage the circuitry in an ethologically relevant manner, is likely critical. For example, opposing heterosynaptic plasticity by LMAN and HVC inputs was only revealed in songbird RA when stimulation patterns mimicked those observed during singing (Mehaffey and Doupe 2015). Moreover, expression of the immediate early gene synaptotagmin IV, which is upregulated in rodent hippocampus during seizures (Vician et al. 1995), is only upregulated within song control regions when birds sing, not when they undergo seizures (Poopatanapong et al. 2006). Fortunately, DREADDs are thought to preserve patterned inputs, either enhancing or damping them, depending on the construct and dose (Roth 2016). Taken together, our results using activation and inhibition of MSNs are internally consistent and provide proof of principle that Area X can exert reciprocal effects to those of LMAN.

Finally, we show that the DREADDs‐induced changes in male song variability fall within the range of social context‐ and practice‐induced changes. Furthermore, under our experimental conditions, naïve female zebra finches can detect DREADDs‐induced alterations and respond to them in a manner consistent with how they responded to naturally occurring changes. Despite this internal consistency, we were surprised that females called more to playbacks of more variable songs, as this is opposite to what Dunning and colleagues found with Bengalese finch females (2014). In that case, females were housed in a colony until use, so could possibly have been sexually experienced, whereas we used sexually naïve females. In the wild, the UD songs of pair‐bonded zebra finches positively correlate with extrapair courtship (Dunn and Zann 1996). Thus, in our case, the calls of naïve female zebra finches to UD song might indicate availability for extrapair copulation. Future work comparing female zebra finch calling to other copulation solicitation displays (as in Dunning et al. 2014) and in experienced versus naïve birds could clarify this issue. The ethologically relevant range of the DREADDs‐induced changes in song, and the fact that DREADDs couple to G‐proteins that naturally exist in MSNS, suggest that the experimental changes induced here capture some of the natural facets of male song and give insight into the manner that such changes in variability endogenously occur.

## Data Accessibility

## Supporting information




**Figure S1.** Viral expression of HSV and CaMKII‐AAV in LMAN and Area X. (A) Viral expression of mCherry signals (red) in LMAN following injection of CaMKII‐AAV into this site. (B) Viral expression of mCherry (red) in LMAN following injection of HSV into this site. Transfection of LMAN projection neurons is evident by the presence of mCherry positive axons in Area X which is demarcated by intense parvalbumin expression (green). (C) The transfection of LMAN projection neurons by CaMKII‐AAV was evident by the overlap of mCherry (arrowheads) with the retrograde labeler fluorogold (blue) injected into Area X. (D) Viral expression of mCherry is widespread within Area X (dashed line) following injection of CaMKII‐AAV into this site. (E–F) Both viral types transfect MSNs as evident by overlap (arrowheads) with the MSN marker FoxP2 (green). (G–H) Both viruses transfect cells in Area X that morphologically resemble MSNs.

**Figure S2.** Intersyllable effects of LMAN manipulations are independent of viral type. LMAN injected birds were separated by viral type. (A–D) Groups of HSV or CaMKII‐AAV injected birds each show the variability injector pattern on measures of intersyllable variability that was seen in combined data.

**Figure S3.** Intrasyllable and syntax effects of LMAN manipulations are dependent on viral type. LMAN injected birds were separated by viral type. (A) HSV injected birds exhibited the intrasyllable variability injector pattern observed in the combined data. (B) HSV injected birds exhibited a syntax entropy variability injector pattern not apparent in the group data. (C) CaMKII–AAV injected birds showed a semblance of a variability injector pattern that was weaker than that of HSV injected birds. (D) No effect on syntax entropy was observed in CaMKII‐AAV injected birds, which may have masked any effect in HSV injected birds.

**Figure S4.** Intersyllable effects of Area X manipulations are independent of viral type. Area X injected birds were separated by viral type. (A–D) Groups of HSV or CaMKII‐AAV injected birds each show the stabilizing effect on intersyllable variability that was seen in combined data.

**Figure S5.** Intrasyllable and syntax effects of Area X manipulations. Area X injected birds were separated by viral type. (A) HSV injected birds showed the stabilizing effect on intrasyllable variability observed in the combined data. (B) No effect was observed on syntax entropy in HSV injected birds. (C) No effect of Area X manipulation on intrasyllable variability was detected in CaMKII‐AAV injected birds (D) No effect on syntax entropy was observed in CaMKII‐AAV injected birds.
