# Supplementary material for: Bidirectional scaling of vocal variability by an avian cortico‐basal ganglia circuit
Source: Physiol Rep. 2018 Apr 24;6(8):e13638. doi: 10.14814/phy2.13638 (PMC5913712; doi:10.14814/phy2.13638)
Supplement: Supplementary file 1 — Figure S1. Viral expression of HSV and CaMKII‐AAV in LMAN and Area X. (A) Viral expression of mCherry signals (red) in LMAN following injection of CaMKII‐AAV into this site. (B) Viral expression of mCherry (red) in LMAN following injection of HSV into this site. Transfection of LMAN projection neurons is evident by the presence of mCherry positive axons in Area X which is demarcated by intense parvalbumin expression (green). (C) The transfection of LMAN projection neurons by CaMKII‐AAV was evident by the overlap of mCherry (arrowheads) with the retrograde labeler fluorogold (blue) injected into Area X. (D) Viral expression of mCherry is widespread within Area X (dashed line) following injection of CaMKII‐AAV into this site. (E–F) Both viral types transfect MSNs as evident by overlap (arrowheads) with the MSN marker FoxP2 (green). (G–H) Both viruses transfect cells in Area X that morphologically resemble MSNs. [file PHY2-6-e13638-s001.pptx]

## Slide 1
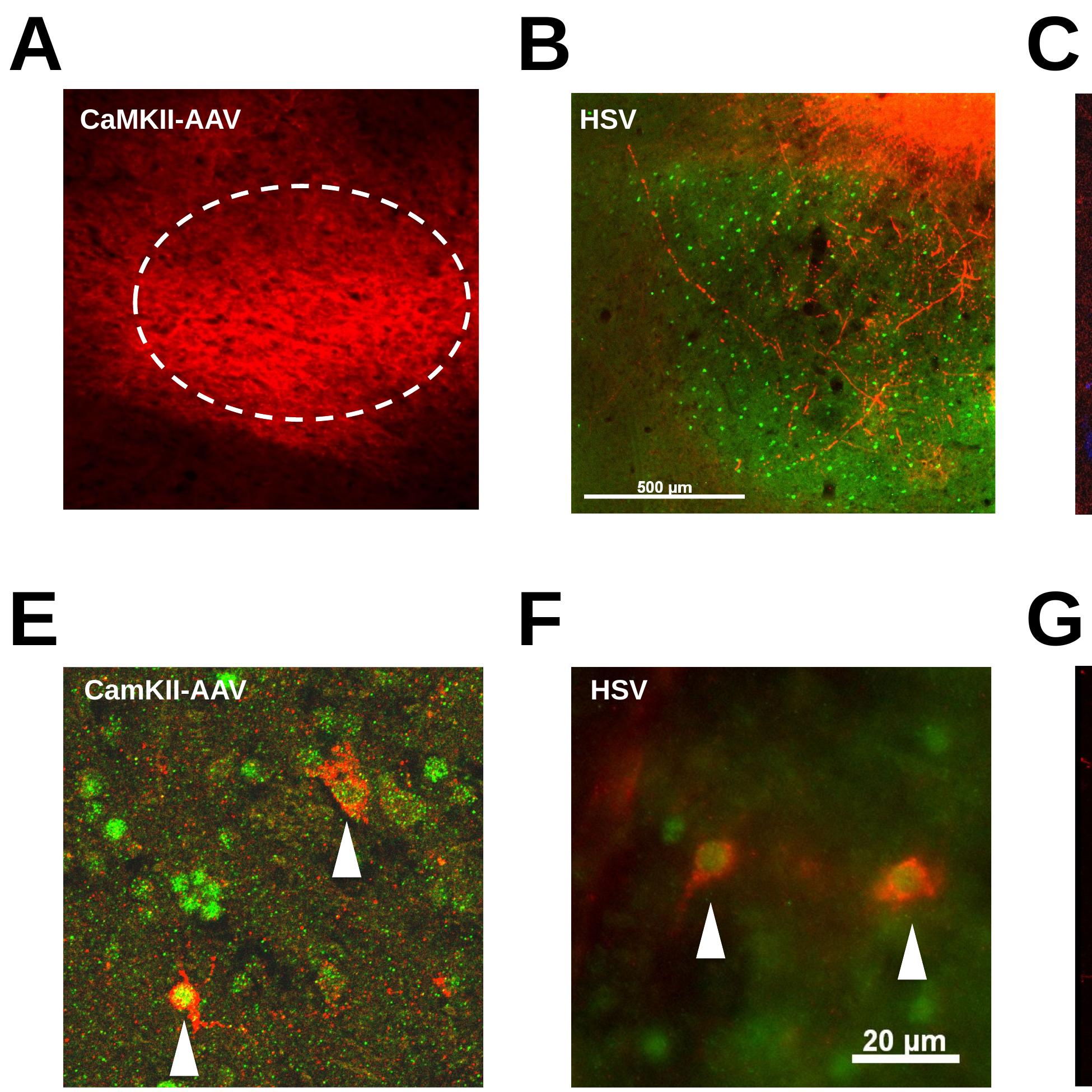

A
B
C
D
CaMKII-AAV
CaMKII-AAV
HSV
HSV-mCherry
CaMKII-AAV1 (Area X)
CamKII-AAV
AAV-eDREADDs3
D
AAV1-CaMKII
E
F
G
H
CamKII-AAV
HSV
HSV
CaMKII-AAV
CaMKII-AAV1
