# Supplementary material for: Bidirectional scaling of vocal variability by an avian cortico‐basal ganglia circuit
Source: Physiol Rep. 2018 Apr 24;6(8):e13638. doi: 10.14814/phy2.13638 (PMC5913712; doi:10.14814/phy2.13638)
Supplement: Supplementary file 2 — Figure S2. Intersyllable effects of LMAN manipulations are independent of viral type. LMAN injected birds were separated by viral type. (A–D) Groups of HSV or CaMKII‐AAV injected birds each show the variability injector pattern on measures of intersyllable variability that was seen in combined data. [file PHY2-6-e13638-s002.pptx]

## Slide 1
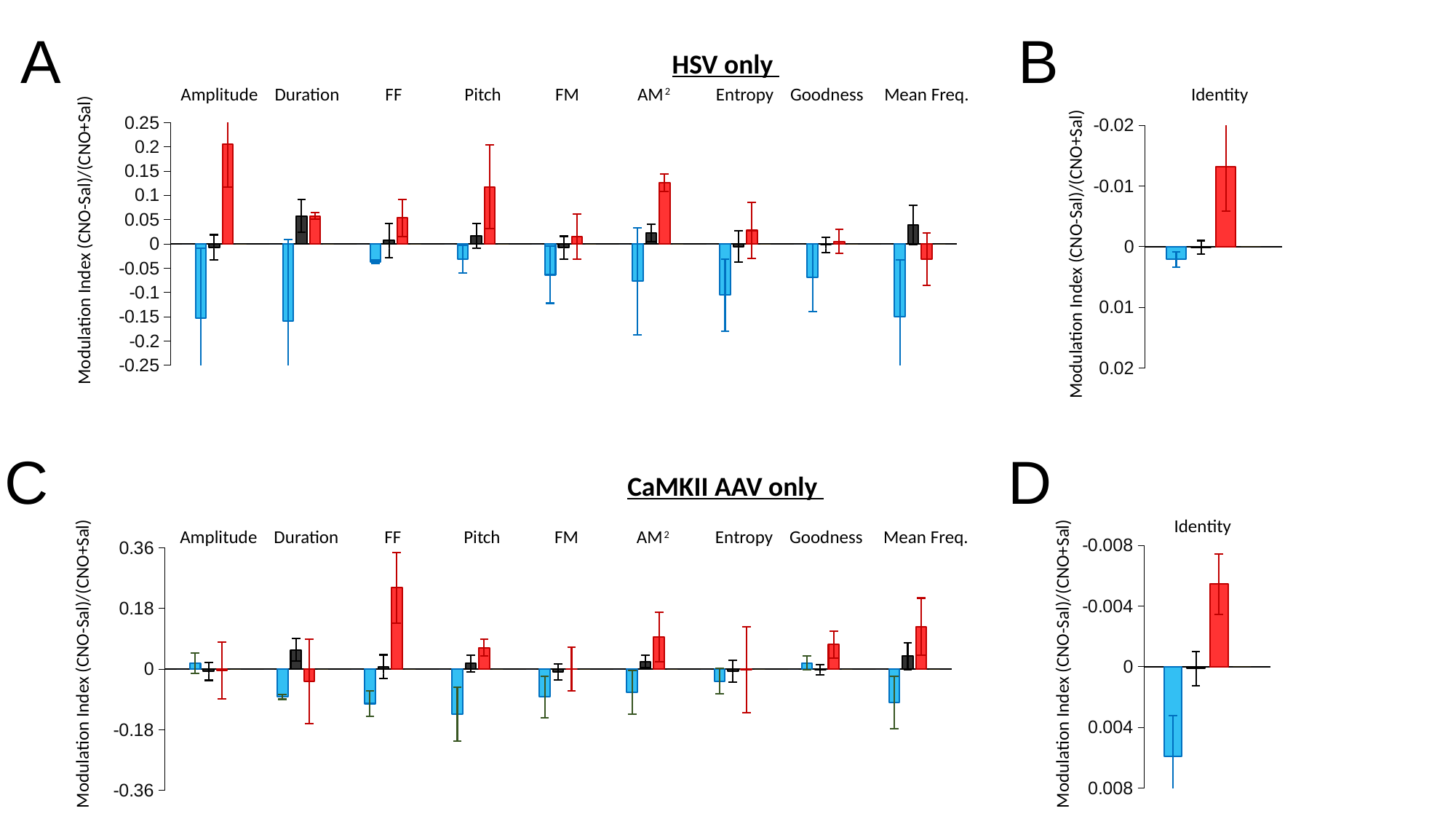

# LMAN – separated by virus; Intersyllable variability
A
B
HSV only
Amplitude Duration FF Pitch FM AM2 Entropy Goodness Mean Freq.
Identity
### Chart
| Category | | | | | |
|---|---|---|---|---|---|
### Chart
| Category | | | | |
|---|---|---|---|---|Modulation Index (CNO-Sal)/(CNO+Sal)
Modulation Index (CNO-Sal)/(CNO+Sal)
C
D
CaMKII AAV only
Identity
Amplitude Duration FF Pitch FM AM2 Entropy Goodness Mean Freq.
### Chart
| Category | | | | |
|---|---|---|---|---|
### Chart
| Category | | | | | |
|---|---|---|---|---|---|Modulation Index (CNO-Sal)/(CNO+Sal)
Modulation Index (CNO-Sal)/(CNO+Sal)
