# Supplementary material for: Bidirectional scaling of vocal variability by an avian cortico‐basal ganglia circuit
Source: Physiol Rep. 2018 Apr 24;6(8):e13638. doi: 10.14814/phy2.13638 (PMC5913712; doi:10.14814/phy2.13638)
Supplement: Supplementary file 3 — Figure S3. Intrasyllable and syntax effects of LMAN manipulations are dependent on viral type. LMAN injected birds were separated by viral type. (A) HSV injected birds exhibited the intrasyllable variability injector pattern observed in the combined data. (B) HSV injected birds exhibited a syntax entropy variability injector pattern not apparent in the group data. (C) CaMKII–AAV injected birds showed a semblance of a variability injector pattern that was weaker than that of HSV injected birds. (D) No effect on syntax entropy was observed in CaMKII‐AAV injected birds, which may have masked any effect in HSV injected birds. [file PHY2-6-e13638-s003.pptx]

## Slide 1
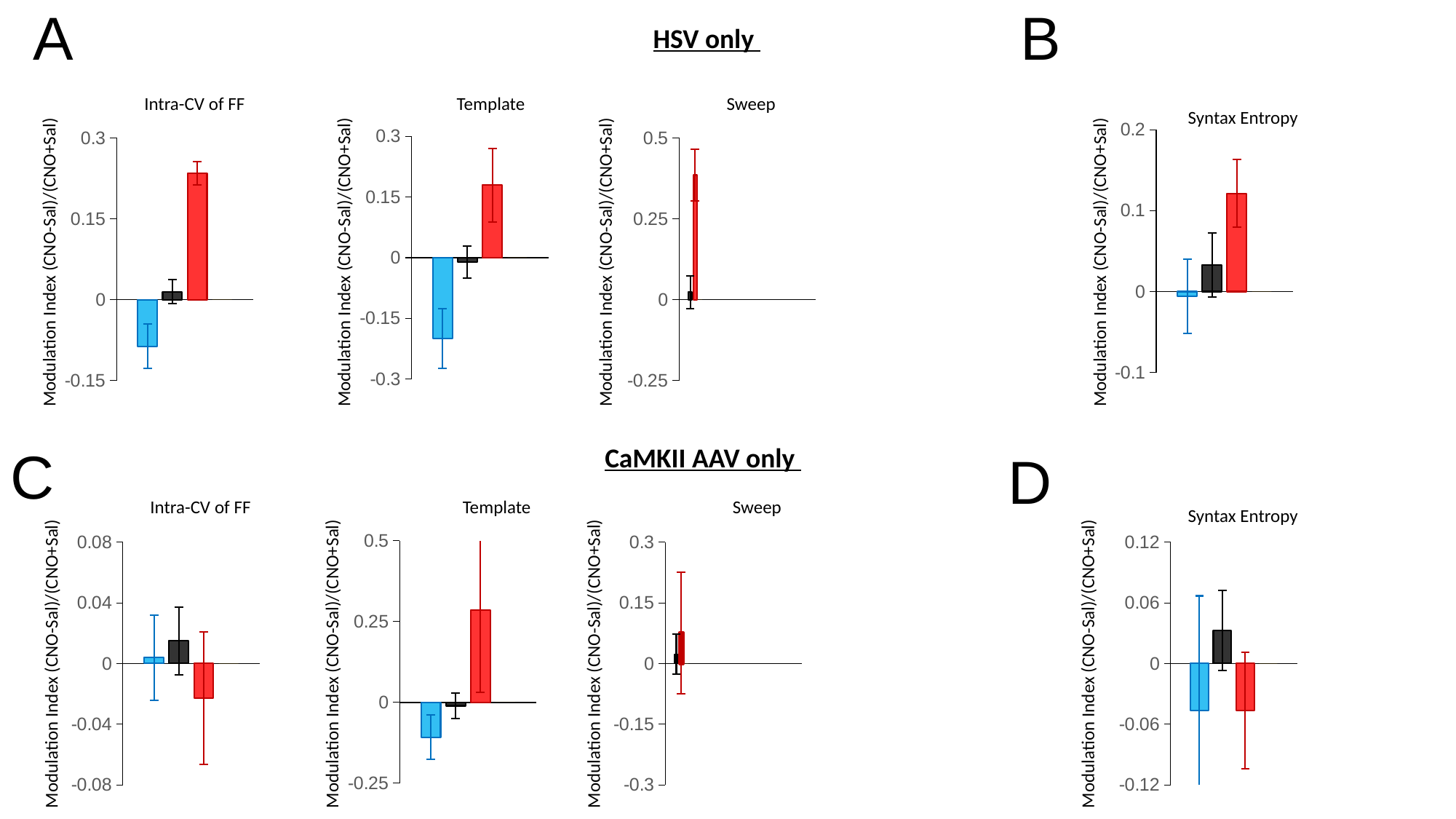

# LMAN– separated by virus; moment-to-moment and syntax variability
A
B
HSV only
Intra-CV of FF
Template
Sweep
### Chart
| Category | | | | |
|---|---|---|---|---|Modulation Index (CNO-Sal)/(CNO+Sal)
### Chart
| Category | | | | |
|---|---|---|---|---|Modulation Index (CNO-Sal)/(CNO+Sal)
### Chart
| Category | | | | |
|---|---|---|---|---|Modulation Index (CNO-Sal)/(CNO+Sal)
### Chart
| Category | | | | |
|---|---|---|---|---|Modulation Index (CNO-Sal)/(CNO+Sal)
Syntax Entropy
C
CaMKII AAV only
D
Intra-CV of FF
Template
Sweep
### Chart
| Category | | | | |
|---|---|---|---|---|Modulation Index (CNO-Sal)/(CNO+Sal)
### Chart
| Category | | | | |
|---|---|---|---|---|Modulation Index (CNO-Sal)/(CNO+Sal)
### Chart
| Category | | | | |
|---|---|---|---|---|Modulation Index (CNO-Sal)/(CNO+Sal)
### Chart
| Category | | | | |
|---|---|---|---|---|Modulation Index (CNO-Sal)/(CNO+Sal)
Syntax Entropy
