# Supplementary material for: Bidirectional scaling of vocal variability by an avian cortico‐basal ganglia circuit
Source: Physiol Rep. 2018 Apr 24;6(8):e13638. doi: 10.14814/phy2.13638 (PMC5913712; doi:10.14814/phy2.13638)
Supplement: Supplementary file 4 — Figure S4. Intersyllable effects of Area X manipulations are independent of viral type. Area X injected birds were separated by viral type. (A–D) Groups of HSV or CaMKII‐AAV injected birds each show the stabilizing effect on intersyllable variability that was seen in combined data. [file PHY2-6-e13638-s004.pptx]

## Slide 1
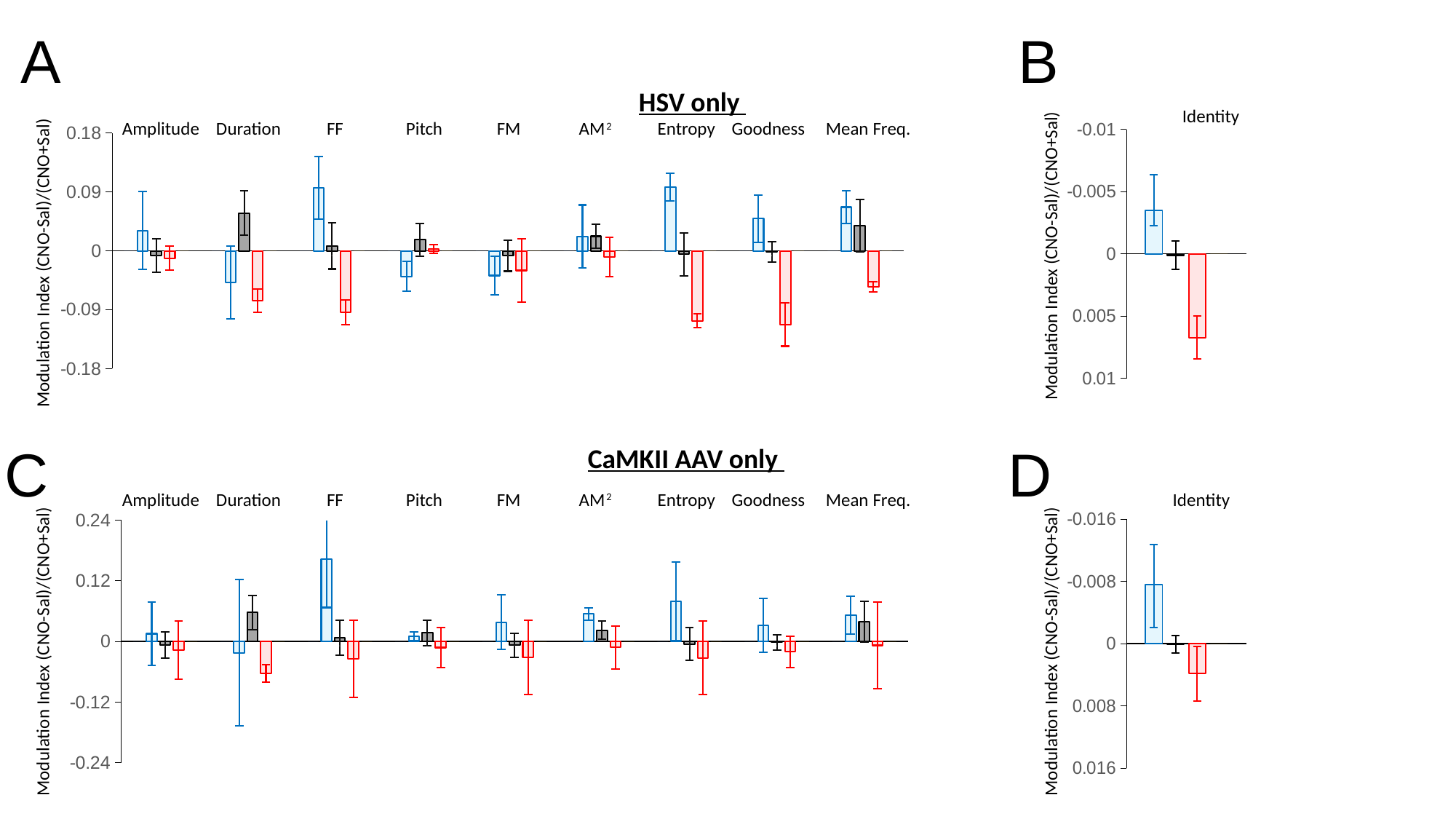

# Area X – separated by virus; Rendition-to-rendition variability
A
B
HSV only
Identity
Amplitude Duration FF Pitch FM AM2 Entropy Goodness Mean Freq.
### Chart
| Category | | | | 0 |
|---|---|---|---|---|
### Chart
| Category | | | | | |
|---|---|---|---|---|---|Modulation Index (CNO-Sal)/(CNO+Sal)
Modulation Index (CNO-Sal)/(CNO+Sal)
C
D
CaMKII AAV only
Identity
Amplitude Duration FF Pitch FM AM2 Entropy Goodness Mean Freq.
### Chart
| Category | | | | | |
|---|---|---|---|---|---|
### Chart
| Category | | | | 0 |
|---|---|---|---|---|Modulation Index (CNO-Sal)/(CNO+Sal)
Modulation Index (CNO-Sal)/(CNO+Sal)
