# Supplementary material for: Bidirectional scaling of vocal variability by an avian cortico‐basal ganglia circuit
Source: Physiol Rep. 2018 Apr 24;6(8):e13638. doi: 10.14814/phy2.13638 (PMC5913712; doi:10.14814/phy2.13638)
Supplement: Supplementary file 5 — Figure S5. Intrasyllable and syntax effects of Area X manipulations. Area X injected birds were separated by viral type. (A) HSV injected birds showed the stabilizing effect on intrasyllable variability observed in the combined data. (B) No effect was observed on syntax entropy in HSV injected birds. (C) No effect of Area X manipulation on intrasyllable variability was detected in CaMKII‐AAV injected birds (D) No effect on syntax entropy was observed in CaMKII‐AAV injected birds. [file PHY2-6-e13638-s005.pptx]

## Slide 1
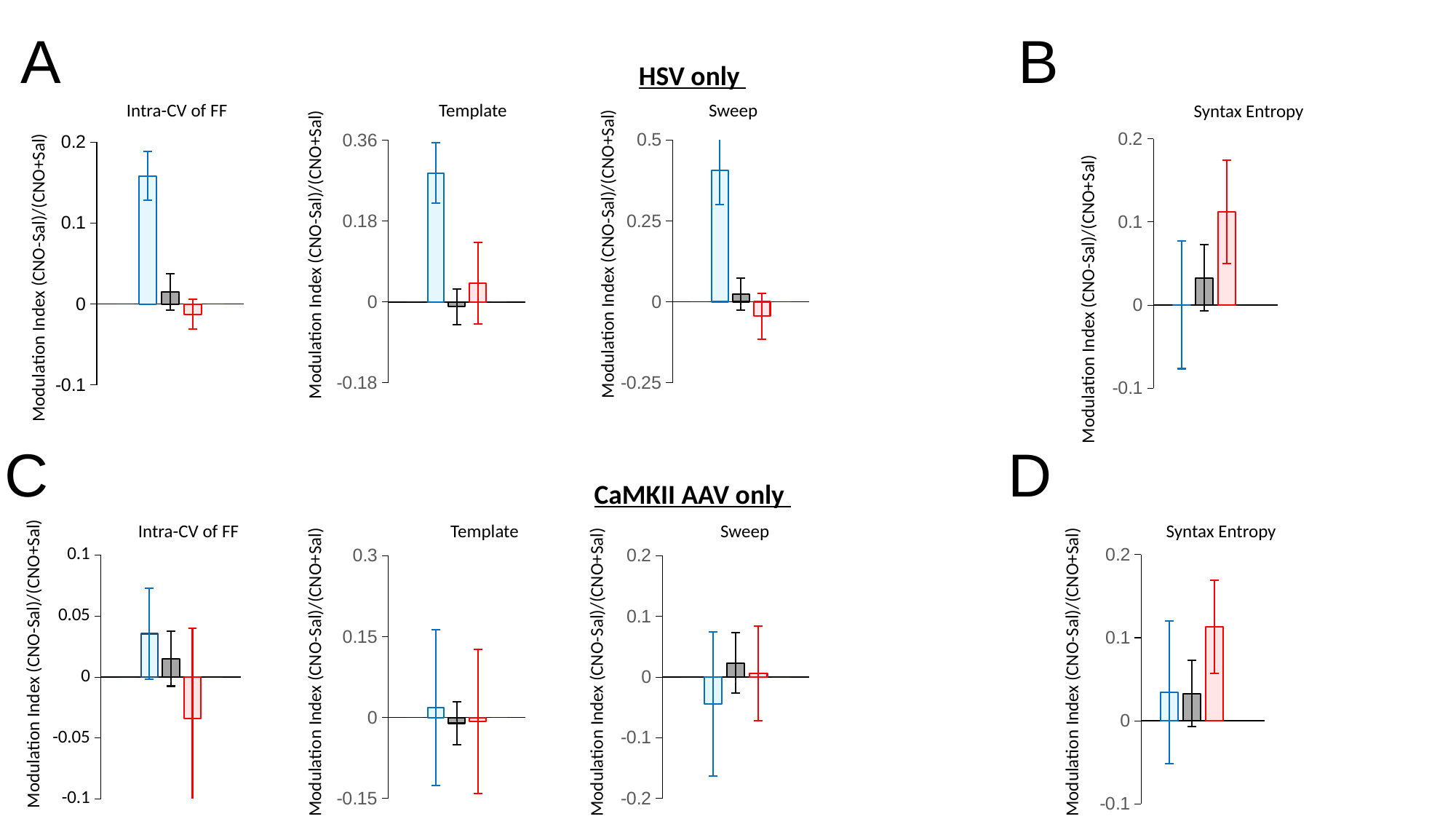

A
B
HSV only
Intra-CV of FF
Template
Sweep
Syntax Entropy
### Chart
| Category | | | | | |
|---|---|---|---|---|---|
### Chart
| Category | | | | |
|---|---|---|---|---|
### Chart
| Category | | | | | |
|---|---|---|---|---|---|
### Chart
| Category | | | | | |
|---|---|---|---|---|---|Modulation Index (CNO-Sal)/(CNO+Sal)
Modulation Index (CNO-Sal)/(CNO+Sal)
Modulation Index (CNO-Sal)/(CNO+Sal)
Modulation Index (CNO-Sal)/(CNO+Sal)
C
D
CaMKII AAV only
Syntax Entropy
Intra-CV of FF
Template
Sweep
### Chart
| Category | | | | | |
|---|---|---|---|---|---|
### Chart
| Category | | | | | |
|---|---|---|---|---|---|
### Chart
| Category | | | | | |
|---|---|---|---|---|---|
### Chart
| Category | | | | |
|---|---|---|---|---|Modulation Index (CNO-Sal)/(CNO+Sal)
Modulation Index (CNO-Sal)/(CNO+Sal)
Modulation Index (CNO-Sal)/(CNO+Sal)
Modulation Index (CNO-Sal)/(CNO+Sal)
#
